# Mass spectrometry-based serum peptidome profiling accurately and reliably predicts outcomes of pemetrexed plus platinum chemotherapy in patients with advanced lung adenocarcinoma

**DOI:** 10.1371/journal.pone.0179000

**Published:** 2017-06-08

**Authors:** Lin Wang, Chuanhao Tang, Bin Xu, Lin Yang, Lili Qu, Liangliang Li, Xiaoyan Li, Weixia Wang, Haifeng Qin, Hongjun Gao, Kun He, Xiaoqing Liu

**Affiliations:** 1 Department of Lung Cancer, Affiliated Hospital of Academy of Military Medical Sciences, Beijing, China; 2 National Center of Biomedical Analysis, Beijing, China; University of Crete, GREECE

## Abstract

**Background:**

Although pemetrexed plus cis/carboplatin has become the most effective chemotherapy regimen for patients with advanced lung adenocarcinoma, predictive biomarkers are not yet available, and new tools to identify chemosensitive patients who would likely benefit from this treatment are desperately needed. In this study, we constructed and validated predictive peptide models using the serum peptidome profiles of two datasets.

**Methods:**

One hundred eighty-three patients treated with first-line platinum-based pemetrexed treatment for advanced lung adenocarcinoma were retrospectively enrolled and randomized into the training (n = 92) or validation (n = 91) set, and pre-treatment serum samples were analyzed using matrix-assisted laser desorption/ionization time-of-flight mass spectrometry (MALDI-TOF-MS) and ClinProTools software. Serum peptidome profiles from the training set were used to identify potential predictive peptide biomarkers and construct a predictive peptide model for accurate group discrimination; which was then used to classify validation samples into “good” and “poor” outcome groups. The clinical outcomes of objective response rate (ORR), disease control rate (DCR), progression-free survival (PFS), and overall survival (OS) were analyzed based on the classification result.

**Results:**

Eight potential peptide biomarkers were identified. A predictive peptide model based on four distinct m/z features (2,142.12, 3,316.19, 4,281.94, and 6,624.02 Da) was developed based on the clinical outcomes of training set patients after first-line pemetrexed plus platinum treatment. In the validation set, the good group had significantly higher ORR (49.1% vs. 8.3%, P <0.001) and DCR (96.4% vs. 47.2%, P <0.001), and longer PFS (7.3 months vs. 2.7 months, P <0.001) vs. the poor group. However, the model did not predict OS (13.6 months vs. 12.7 months, P = 0.0675).

**Conclusion:**

Our predictive peptide model could predict pemetrexed plus platinum treatment outcomes in patients with advanced lung adenocarcinoma and might thus facilitate appropriate patient selection. Further studies are needed to confirm these findings.

## Introduction

Currently, adenocarcinoma is the most common histological subtype of lung cancer, the leading cause of cancer-related deaths worldwide [1]. Despite great progress in targeted and immune therapies, chemotherapy remains the standard treatment for lung cancer. The American Society of Clinical Oncology suggests that patients with advanced non-squamous non-small cell lung cancer (NSCLC) patients who are not suitable for targeted therapy or immunotherapy should receive a platinum-based combination of two cytotoxic drugs [2]. In this context, pemetrexed is one of the most effective agents when combined with cisplatin or carboplatin [3,4]. However, a previous report stated a pooled objective response rate (ORR) and median progression-free survival (PFS) of 37.8% and 5.7 months, respectively, for platinum-based pemetrexed chemotherapy, indicating that many patients do not respond to this regimen [5]. Furthermore, unlike targeted or immune therapies, there are no clinical biomarkers to indicate which patients would benefit from pemetrexed or platinum-based chemotherapy. Recently, thymidylate synthase (TS) and excision repair cross-complementation group 1 (ERCC1) showed promise as predictive biomarkers for pemetrexed and platinum-based agents, respectively; however, these biomarkers must be assessed in tumor tissues and have not been validated prospectively in patients with lung adenocarcinoma [6,7]. Furthermore, a single predictive biomarker strategy is unrealistic because pemetrexed and platinum are usually administered in combination. Accordingly, new treatment selection tools are desperately needed to enhance the efficacy of this important regimen.

Recently, proteomic/peptidomic analyses, which complement genetic analyses, have become integral to investigations of tumor biology [8]. In addition, protein/peptide signatures can be tested using serum samples and may more accurately characterize the disease characteristics and development. Serum marker classification models yield patterns of multiple serum biomarkers, which provide better sensitivity and discrimination relative to a single biomarker. Many studies have tested the ability of proteomics/peptidomics strategies to facilitate early tumor detection and identify patients who would benefit from specific targeted therapies [9–14]. However, this method has not previously been used to predict chemotherapeutic outcomes.

Of the various proteomic/peptidomic techniques, matrix-assisted laser desorption/ionization time-of-flight (MALDI-TOF) mass spectrometry (MS) is uniquely suited to the analysis of complex biological samples [15]. MALDI-TOF MS devices comprise three main components: an ion source to ionize molecules and transfer them into a gas phase, a mass analysis device that separates molecules according by mass, and a detector that monitors the separated ions. During MALDI-TOF MS analysis, the separation of molecules by mass facilitates the creation of a mass spectrum characterized by ion masses and intensities. Previously, MALDI-TOF MS and bioinformatics have been combined to develop diagnostic or predictive models that could eventually be used in clinical practice [12,16–18].

In this study, we used weak cation exchange magnetic beads coupled with MALDI-TOF MS to obtain serum peptidome profiles of a training dataset, which were then used to construct a predictive peptide model. The discriminative ability of this model was then tested using a validation dataset. Specifically, we used the ORR, DCRPFS, and OS to assess the predictive value of our predictive classification model.

## Materials and methods

### Patients and samples

**Fig 1:**
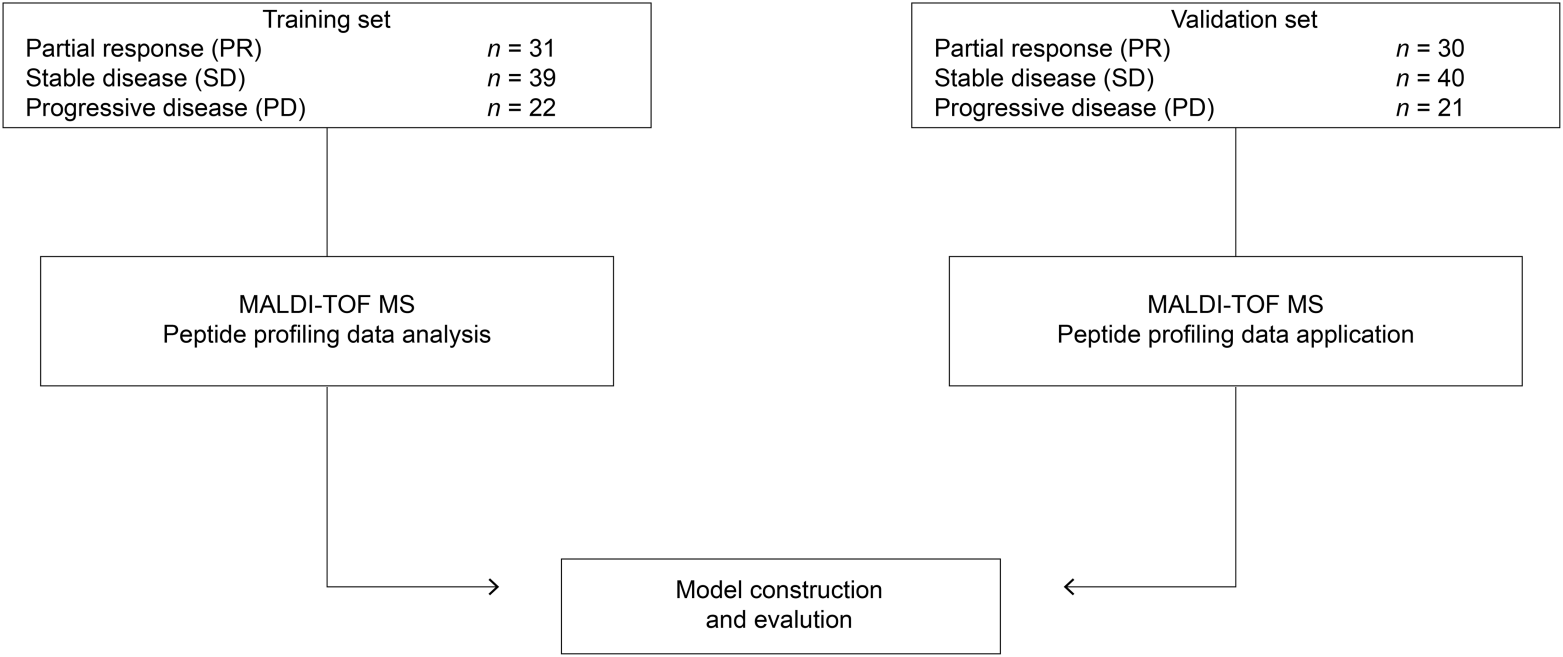
Study overview. This flowchart describes the construction and testing of serum-based predictive peptide models for patients with advanced lung adenocarcinoma who were treated with first-line pemetrexed plus platinum-based chemotherapeutic regimens.

A study overview is provided in Fig 1. A total of 183 patients with advanced lung adenocarcinoma who were treated with first-line pemetrexed plus platinum at the Affiliated Hospital of Academy of Military Medical Sciences from December 2012 to November 2014 were enrolled in this retrospective study. The eligibility criteria included a confirmed new diagnosis of advanced lung adenocarcinoma (stage IIIB or stage IV), no history of prior chemotherapy or targeted therapy, good organ function, and an Eastern Cooperative Oncology Group performance status of 0–2.

All patients received at least two cycles of pemetrexed plus cisplatin (500 mg/m^2^ and 75 mg/m^2^, respectively) or carboplatin (area under the curve: 5). Tumors were assessed at baseline using computed tomography, and the same radiological assessment was repeated every two cycles to assess the disease status. Disease response and progression were assessed using the Response Evaluation Criteria in Solid Tumors, version 1.0. Patients whose lesions disappeared during treatment were classified as having achieved a complete response (CR). Patients with a ≥30% decrease in the target lesion size were classified as having achieved a partial response (PR). Patients with a change in lesion size ranging from an increase of <20% to a decrease of <30% and no new lesions were classified as having stable disease (SD). Patients with an increase of ≥20% in lesion size or with new lesions were classified as having progressive disease (PD). The ORR was defined as the sum of the CR and PR rates, and the disease control rate (DCR) was defined as the sum of the ORR and SD rate.

PFS was defined as the time interval from the start of treatment to disease progression. OS was defined as the time from the date of diagnosis to the date of death or the last follow-up date (November 20, 2016). The smoking status was determined from patients’ medical records, and those who had smoked >100 cigarettes in lifetime were considered smokers. Pre-treatment (baseline) serum samples were obtained from consenting patients and collected in vacuum blood collection tubes containing coagulant and separation gel, separated via centrifugation (10 min at 4,000 rpm, 4°C), and stored at –80°C until analysis.

This study’s retrospective design was approved by the ethics committee of Affiliated Hospital of Academy of Military Medical Sciences (approval #2012-11-171). All patients provided informed consent to receive treatment and for the testing of their serum samples.

### Study population and outcomes

Responses to chemotherapy were classified as CR, PR, SD and PD. As the sex ratio was imbalanced in favor of male patients, all patients were stratified and randomized into the training and validation sets by sex and treatment response before the analyses to balance the influences of these factors. The training group was used to develop peptide models that could discriminate patients who would and would not benefit from treatment. The validation group was then used to test the predictive power of the model derived from the training set.

Patients in the training group were divided into four clinical groups: PD (disease progression at ≤1.5 months), short SD (disease stability for ≤3 months), long SD (disease stability for >3 months), and PR. To optimize classification parameters, representative spectra of each clinical group were selected from among the training group. Good clinical outcomes included a CR, PR, or long SD, whereas and poor clinical outcomes included PD or short SD.

### Sample preparation and mass analysis (peptide profiling)

Serum samples were thawed on ice and fractionated using weak cation exchange magnetic beads (MB-WCX, YiXin Bochuan Bio-Technique Co. Ltd., Beijing, China) before MS analysis, which was conducted from November 25 to December 5, 2016. These magnetic beads, which exhibited good peptide-capturing performance, were used to fractionate serum samples according to the manufacturer’s instructions. We added 5 μL of the serum sample to a pre-mixture of 10 μL of binding solution and 7 μL of MB-WCX beads in a polymerase chain reaction tube. The solution was intensively mixed, incubated for 5 min, and placed on a magnetic separator to isolate the unbound solution. The bound peptides were eluted from the magnetic beads after two rounds of bead separation and washing. Finally, 1 μL of the peptide eluate was mixed with 1 mL of MALDI-TOF matrix (a saturated solution of 4-hydroxy-3,5-dimethoxycinnamic acid in 50% acetonitrile with 0.5% trifluoroacetic acid), which was then spotted onto the sample anchor spots of an AnchorChip target plate (Bruker Daltonics Inc., Bremen, Germany). The MALDI-TOF MS analyses were performed on an Ultraflex III MALDI-TOF MS device (Bruker Daltonics Inc.) with the following settings: linear positive ion mode, repetition rate of 200 Hz, ion source voltages of 25 kV and 23.50 kV, lens voltage of 6.5 kV, pulsed ion extraction time of 100 ns, and nitrogen pressure of 1,700–2,000 mbar. All signals with a signal-to-noise ratio of >5 in a mass range of 800–10,000 Da were recorded using FlexAnalysis software (version 3.4; Bruker Daltonics Inc.). The peptidomic patterns and models were processed using ClinPro Tools bioinformatics software (version 3.0; Bruker Daltonics Inc.).

### Data processing and statistical analysis

The following workflow was used for the data processing and analysis with FlexAnalysis and ClinPro Tools software: each spectrum was normalized to its total ion current and recalibrated according to the prominent and common m/z values, after which the baseline was subtracted, the peaks were smoothed before detection, and the peak areas were calculated for each spectrum. All peak signals were processed for noise reduction using a top-hat baseline in the 800–10,000 Da range. For the peptide peaks, the expressions of the same mass-to-charge ratios (m/z) were compared between the good and poor response groups using parametric testing (t test); we also tested whether these peptide patterns could be classified.

During model construction, we used only the spectra and clinical outcome data of the training set. Three algorithms (genetic algorithm [GA], supervised neural networks [SNN], and quick classifier [QC]) were used to establish the prediction models. Next, each model was applied to the validation set to test its ability to identify patients with good and poor responses. The validation process was performed in a blinded manner, and samples that had been classified prior to obtaining clinical outcome data were available to the investigators.

ORR and DCR outcomes were compared using the chi-square test or Fisher's exact test, whereas and PFS and OS were analyzed using the Kaplan–Meier method and the log-rank test. Survival outcomes were reported as median durations with 95% confidence intervals (CIs). Multivariable Cox proportional hazard analyses were performed to evaluate the relevance of various clinical features. All statistical tests were two-tailed, and a P-value of <0.05 was considered statistically significant. All statistical tests were performed using SPSS software (version 19; SPSS Inc., Chicago, IL, USA).

## Results

### Patient characteristics

The clinical characteristics of the training and validation groups are presented in Table 1 and S1 Dataset (DOI: 10.6084/m9.figshare.5043280). Of the 183 patients included in this study, 92 and 91 were assigned to the training and validation groups, respectively. Most patients had stage IV disease and an Eastern Cooperative Oncology Group performance status of 1. All patients had histologically diagnosed adenocarcinoma and received first-line pemetrexed plus cis/carboplatin treatment.

**Table 1:** The clinical characteristics of the enrolled patients.

Characteristics	Training set (n = 92)	Validation set (n = 91)
Age (years)		
Median	56	57
Range	37–75	33–78
Sex, No. (%)		
Male	57 (62.0)	57 (62.7)
Female	35 (38.0)	34 (37.3)
Disease stage, No. (%)		
IIIB	11 (12.0)	7 (7.7)
IV	81 (88.0)	84 (92.3)
Histologic type, No. (%)		
Adenocarcinoma	92 (100)	91 (100)
Smoking History, No. (%)		
Never	45 (48.9)	43 (47.2)
Former or Current	47 (51.1)	48 (52.8)
Metastases, No. (%)		
Brain	12(13.1)	20(22.0)
Bone	26(28.3)	28(13.3)
RECIST, No. (%)		
Complete response	0 (0)	0 (0)
Partial response	31 (33.7)	30 (33.0)
Stable disease-long	25 (27.2)	27 (29.7)
Stable disease-short	14 (15.1)	13 (14.2)
Progressive disease	22 (24.0)	21 (23.1)
EGFR gene mutation status, No. (%)		
E19del	5 (5.4)	7 (7.7)
L858R	5 (5.4)	5 (5.5)
Wild-type	44 (47.8)	35 (38.5)
Unknown	38 (41.4)	44 (48.3)
Treatment, No. (%)		
Pemetrexed +cisplatin	72 (77.2)	72 (79.1)
Pemetrexed +carboplatin	20 (22.8)	19 (20.9)

Abbreviations: RECIST, Response Evaluation Criteria in Solid Tumors; E19del, exon 19 deletion; L858R, exon 21 (L858R) mutation

### Comparison of peptidomic data

**Fig 2:**
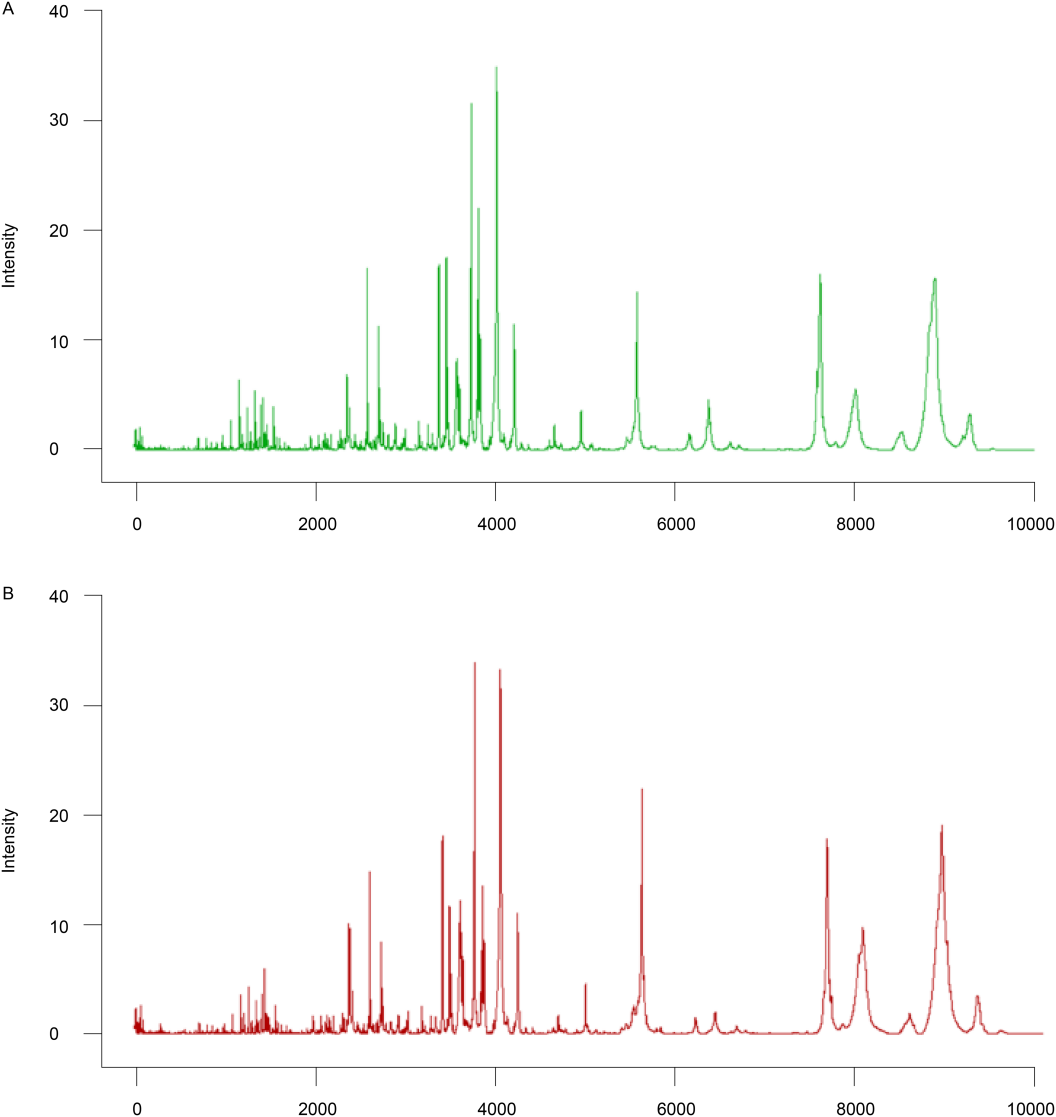
Average serum peptide fingerprints. (2A) Peptide profile of the good outcome group (green, n = 56) generated using ClinPro Tools. (2B) Peptide profile of the poor outcome group (red, n = 36) generated using ClinPro Tools. x-axis, mass to charge ratio; y-axis, relative intensity.

**Fig 3:**
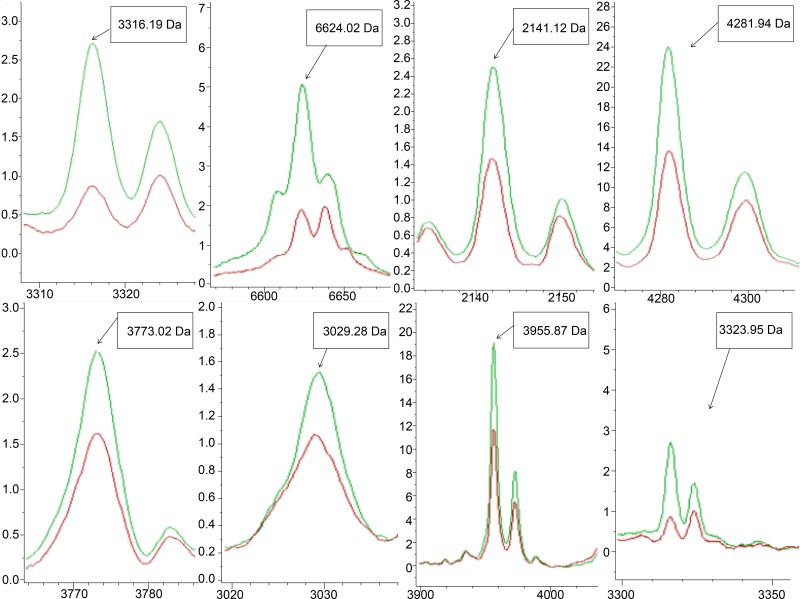
Significantly expressed mass peaks. Eight peaks were significantly differentially expressed between the good outcome (green line) and poor outcome (red line) groups. x-axis, mass to charge ratio; y-axis, relative intensity.

We compared peptidome MS data from the good and poor responders in the training set. A total of 136 peptide peaks were detected; of these, eight peaks differed significantly between the good and poor outcome groups. Patients who achieved good clinical outcomes exhibited highly expressed mass spectra of m/z 3316.19, 6624.02, 2142.12, 4281.94, 3773.02, 3029.28, 3955.87, and 3323.95 Da (Table 2 and Figs 2 and 3).

**Table 2:** The eight mass peaks significantly differentially expressed in the training set.

MASS[Da]	p-value^1^	Good outcome group	Poor outcome group	Identified peptide sequence	Identified proteins
3316.19	<0.000001	3.09 ± 0.92	1.18 ± 0.42	K.NGVDGVYSADPNKDASAVK FDTLTHLDIINK.G	uridylate kinase
2142.12	0.00195	2.83 ± 1.23	1.73 ± 1.18	K.AVEYYFASDASAVIEHTNR.V	Glucosamine--fructose-6-phosphate aminotransferase
6624.02	<0.000001	7.38 ± 4.25	2.73 ± 2.05	Unknown	
4281.94	0.00255	24.8 ± 12.31	14.24 ± 11.75	Unknown	
3773.02	0.0126	2.77 ± 1.45	1.82 ± 1.11	Unknown	
3029.28	0.0126	1.76 ± 0.72	1.32 ± 0.46	Unknown	
3955.87	0.0253	19.68 ± 11.63	12.22 ± 9.91	Unknown	
3323.95	0.0493	2.09 ± 1.5	1.34 ± 0.87	Unknown	

^**1**^ Calculated using the t-test or Wilcoxon test.

### Construction of predictive peptide models in the training set

The ClinPro Tools 3.0 software package (Bruker Daltonics) was used to analyze all serum sample data derived from the training set. The processed data were then used for visualization and statistical analysis. Statistically significant differences in peptide quantities were determined using Welch’s *t*-test at a significance level of *P* < 0.05. Data from the training set were subjected to three different mathematical model algorithms: the Genetic Algorithm (GA), Supervised Neural Network (SNN), and Quick Classifier (QC). We assessed the performances of these three models by considering the cross-validation and recognition capability. Finally, the QC algorithm was used to set a prediction model, and a peptidome pattern classification was constructed. This model was based on four significantly different peaks at m/z 2,142.12, 3,316.19, 4,281.94, and 6624.02 Da, which provided a recognition capability of 94.74% and cross-validation value of 91.74% (Table 3). This classification model correctly identified 100% of patients with poor outcomes and 83.47% of patients with good outcomes in the training set.

**Table 3:** Included peptide peaks and performances of predictive classification models.

	Genetic algorithm	Supervised Neural Network	Quick Classifier
MASS [Da]	3773.02	3316.19	2142.12, 3316.19, 4281.94, 6624.02
Cross validation	60.17%	86.97%	91.74%
Recognition	73.26%	93.39%	94.74%

### Blind testing of the predictive peptide model in the validation set

**Fig 4:**
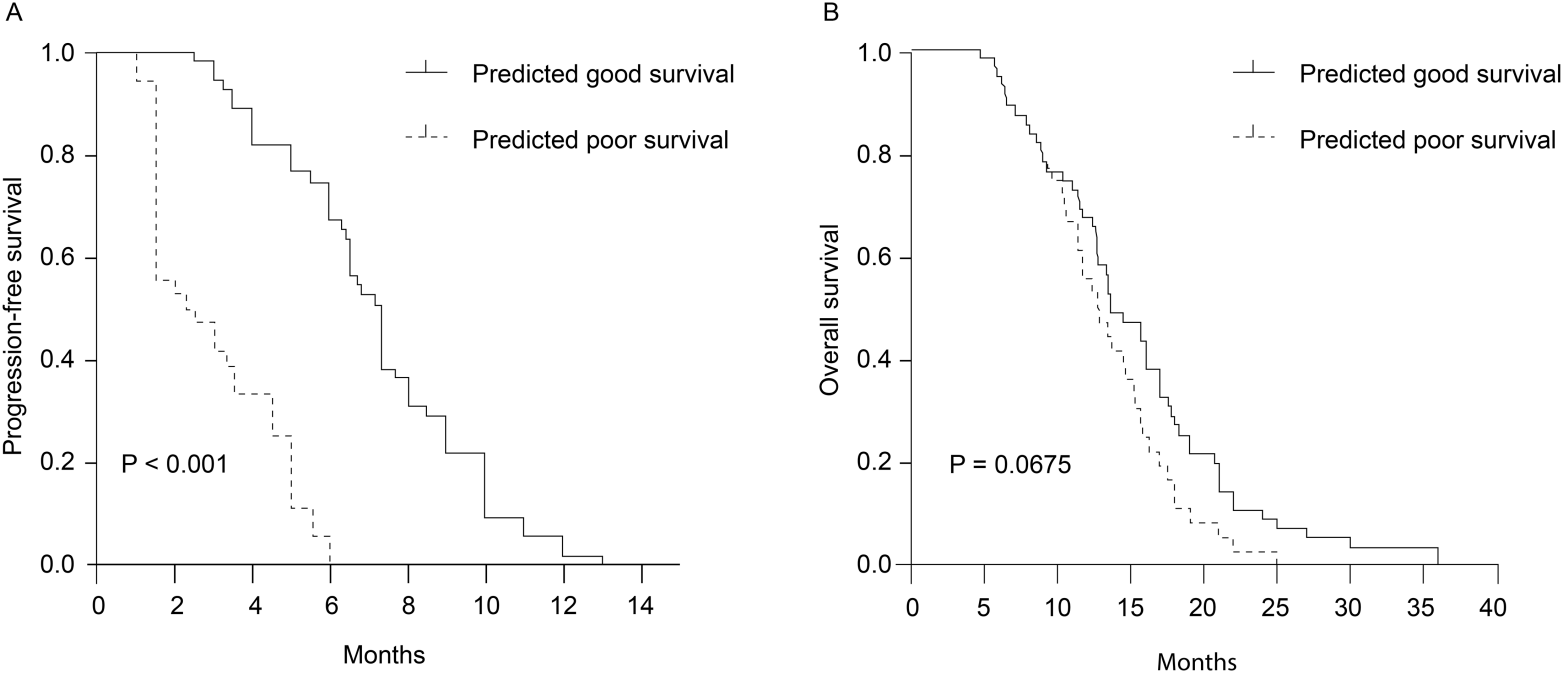
Kaplan–Meier survival analysis. (4A) Progression-free survival according to baseline classification results in the validation set (7.3 months [95% confidence interval (CI): 6.735–7.865 months] vs. 2.7 months [95% CI: 0.939–4.461 months], P <0.001). (4B) Overall survival according to baseline classification results in the validation set (13.6 months [95% CI: 11.109–16.091 months] vs. 12.7 months [95% CI: 10.201–15.199 months], P = 0.0675).

Once the peptide prediction model was established with all parameters frozen, it was tested in the validation set of 91 patients in a blinded manner. All the samples were successfully classified as chemo good (n = 55, 60.4%) or chemo poor (n = 36, 39.6%). In other words, the model identified 55 and 36 patients as having achieved good and poor responses, respectively. When we compared the ORR, DCR, PFS, and OS of these two groups, we found that the chemo good group exhibited a significantly higher ORR (49.1% vs. 8.3%, P <0.001) and DCR (96.4% vs. 47.2%, P <0.001) and significantly longer PFS (7.3 months [95% CI: 6.735–7.865 months] vs. 2.7 months [95% CI: 0.939–4.461 months], P <0.001). However, the peptide model was not predictive of OS (13.6 months [95% CI: 11.109–16.091 months] vs. 12.7 months [95% CI: 10.201–15.199 months], P = 0.0675) (Table 4 and Fig 4).

**Table 4:** Results of model-based classification in the validation set.

	Classified as “good survival”	Classified as “poor survival”	Total	Sensitivity	Specificity	Accuracy
PR	27	3	30	89.5%	88.2%	89%
SD	26	14	40			
PD	2	19	21			
Total	55	36	91			

Abbreviations: PR, partial response; SD, stable disease; PD, progressive disease.

### Multivariable analysis of the validation set

Cox multivariable analyses of PFS according to classification results, age, sex, and smoking status were performed using validation group data. The analyses demonstrated that only the classification results were independently associated with a PFS benefit, as patients who achieved good responses had a significantly lower risk of progression (hazard ratio [HR]: 0.124, 95% CI: 0.067–0.227, log-rank P <0.001).

### Identification of peptide peaks

**Fig 5:**
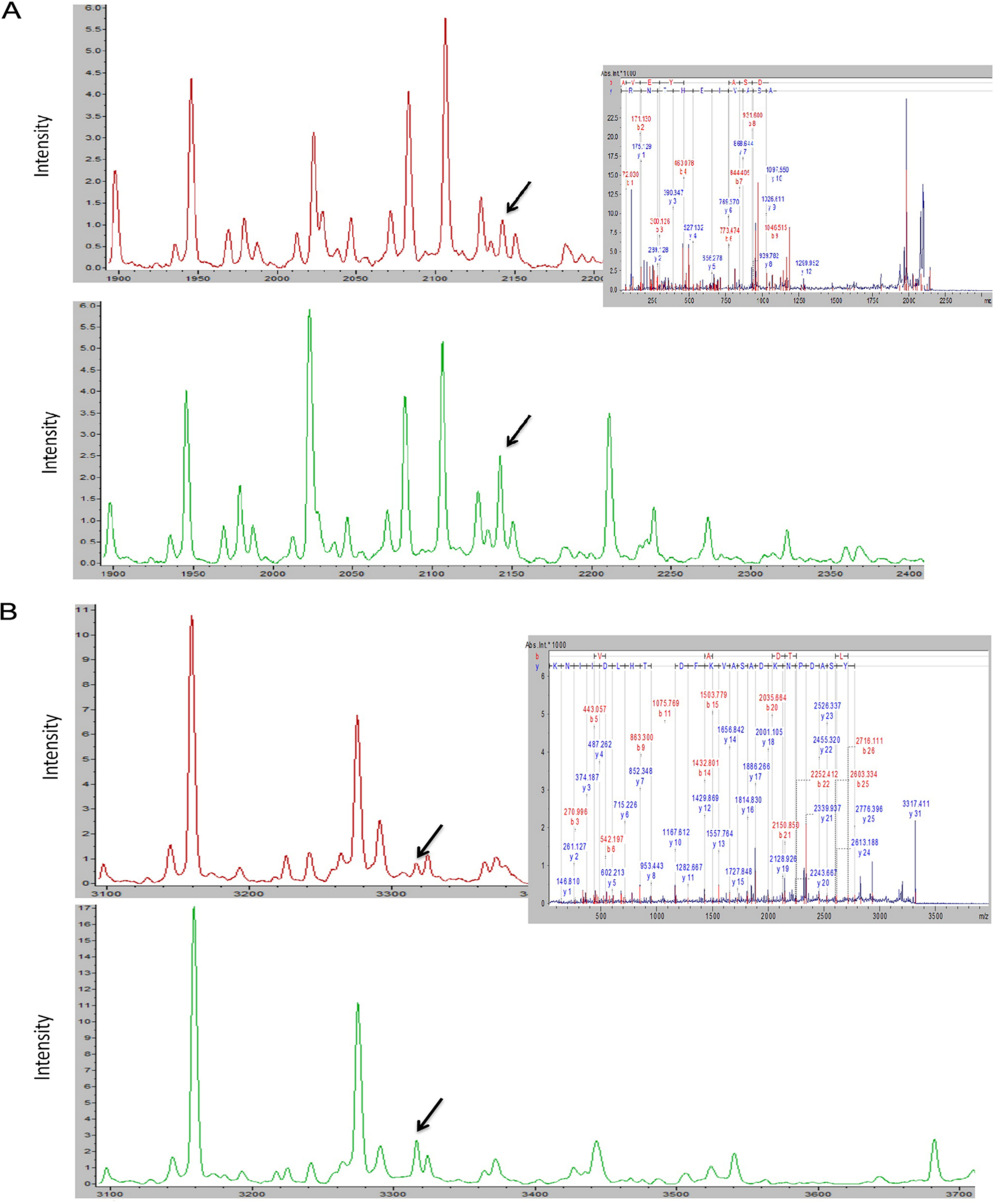
Identification of two differentially expressed serum peptides. Purified peptides from the poor outcome (red line) and good outcome (green line) group were sequenced using LTQ-Orbitrap-MS/MS. 5A and 5B present the fragment ion spectra of the sequences K.AVEYYFASDASAVIEHTNR.V and K.NGVDGVYSADPNKDASAVKFDTLTHLDIINK.G, respectively.

The LTQ Orbitrap-MS/MS successfully identified two of the eight peptides that had been differentially expressed between the good and poor outcome subgroups in the training set. All eight peaks were down-regulated in the poor outcome subgroup, and an MS/MS analysis of two down-regulated peaks, 2,142.12 Da and 3,316.19 Da, revealed respective sequences of K.AVEYYFASDASAVIEHTNR.V and K.NGVDGVYSADPNKDASAVKFDTLTHLDIINK.G. These sequences corresponded to fragments of glucosamine-fructose-6-phosphate aminotransferase and uridylate kinase, respectively (Table 2 and Fig 5).

## Discussion

In this study, we combined magnetic bead-assisted serum peptide capture with MALDI-TOF MS to compare the peptidomic profiles of patients who exhibited different clinical outcomes in response to first-line pemetrexed plus platinum-based chemotherapy for advanced lung adenocarcinoma. Our results indicated that our novel, serum peptide pattern-based prediction model was useful for discriminating patients who would benefit from this regimen. To the best of our knowledge, this is the first attempted use of this methodology to predict the clinical outcomes of pemetrexed plus platinum-based chemotherapy in this patient population.

Although targeted therapies and immune checkpoint inhibitors have significantly improved the outcomes of selected patients with non-small cell lung cancer, chemotherapy remains the mainstay of treatment for thousands of lung cancer patients worldwide [19–21]. Unfortunately, chemotherapy is still administered clinically using a “one-size fits all” approach. Accordingly, predictive biomarkers are desperately needed to identify chemosensitive patients and select appropriate drug combinations, thus avoiding unnecessary toxicities and costs and eventually improving patients' outcomes. To date, two molecules, ERCC1 and TS, have been identified as potential biomarkers [6,7,22]. However, biomarker studies have been hindered by the frequent administration of combinations of chemotherapeutic agents. Meanwhile, the availability and heterogeneity of tumor samples continue to present challenges to clinicians who must select patients [23]. Therefore, novel, noninvasive patient selection tools used to develop predictive models of treatment outcomes must better integrate multiple markers.

The human low molecular weight (1–10 KDa) serum peptidome includes many cytokines, peptide hormones, endogenous peptide products, and protein fragments, some of which may be uniquely suited for diagnostic, prognostic, or predictive peptide biomarker discovery [18,24–28]. In other words, the serum peptidome contains an enormous wealth of unexplored biomarker information. Multiple biomarkers or biomarker patterns are now widely recognized as useful clinical tools, and various proteomic approaches have been applied to biological fluid-based biomarker discovery. Moreover, MALDI-TOF MS has exhibited good performance in the low-molecular-mass range [16] and can detect low molecular weight peptides at high levels of sensitivity and resolution, and is therefore considered a useful and standard method for serum peptidome profiling in many diseases [29]. In the present clinical proteomics study, we advantageously combined MALDI-TOF profiling combined with bioinformatics (FlexAnalysis and ClinPro Tools in this study) to the discovery of peptide biomarker patterns in a human disease.

However, the co-existence of highly abundant proteins and other factors in human serum makes it difficult to directly analyze the blood peptidome. Accordingly, targeted peptide enrichment is required [30]. One option for complex sample management is the elimination of the most abundant proteins. However, this depleted protein fraction may contain important disease biomarkers [31]. In 2007, Fiedler et al. revealed that MB-MALDI-TOF MS (magnetic bead-based fractionation flowed by MALDI-TOF MS) is likely to be more sensitive than SELDI-TOF MS for some later generated peaks [32]. Magnetic beads have been developed to purify and fractionate the proteome in serum, as well as in other body fluids. Accordingly, the MB-WCX (weak cation exchange magnetic beads) peptide profiling kit was developed and used to enrich serum peptides, as well as low molecular weight peptides (1–10 KDa), prior to MALDI-TOF MS analysis. Many studies have confirmed that magnetic bead fractionation plus MALDI-TOF MS is a highly sensitive and reproducible approach to serum profiling in different cancers [9,10,33]. To ensure reproducibility in the present study, the same researcher fractioned all serum samples using MB-WCX, and MALDI-TOF MS analyses were performed on the same day. ClinPro Tools 3.0 software was then used to analyze serum peptidome profiles and establish a predictive peptide model with high levels of cross-validation and recognition that could accurately distinguish patients with good or poor outcomes after pemetrexed plus platinum-based chemotherapy.

To eliminate potential influences of previous treatments, we only included previously untreated patients who had received first-line pemetrexed plus platinum-based chemotherapy, as Lazzari et al. reported that serum peptidome profiles could change during the course of treatment [34]. We also collected pre-treatment serum samples, as we inferred that these might allow us to more accurately evaluate the original disease state, a critical component of model construction, and avoid changes induced by chemotherapy.

Previous studies demonstrated that algorithm-based models generated using similar methods could identify patients who were sensitive to epidermal growth factor receptor tyrosine kinase inhibitors (EGFR-TKIs) [12,24–26]. Unlike those studies, however, we combined the response and PFS to optimize our classification algorithm (rather than either outcome alone). We classified patients in the training set as having good or poor responses in an attempt to provide broad coverage of all patients, and identified eight potential biomarkers that distinguished these patients through our exploration of inter-group peptidome differences. Although three algorithms were used to develop a predictive classification model, our final successful predictive model was based on four peptides and a QC algorithm that could discriminate between patients with good and poor outcomes.

In the validation set, our predictive model identified chemosensitive patients, or those classified in the chemo good, as having a significantly higher ORR and DCR and significantly longer PFS relative to the chemo poor group. Cox multivariable analyses also confirmed that only these classification results correlated independently with a PFS benefit. Most patients with a PR (27/33) or PD (19/21) were classified correctly, thus yielding a high level of accuracy. Moreover, patients with long SD were also distinguished from those with short SD. The study results suggest that patients with a poor classification might exhibit primary resistance to pemetrexed plus platinum-based chemotherapy. Although the predictive peptide model did not predict OS outcomes, we cannot exclude a prognostic role of this peptide model among patients receiving the designated chemotherapy regimen. To confirm whether the predictive power of our peptide model is specific to a pemetrexed plus platinum-based regimen, cohorts of patients treated with the targeted therapy or other chemotherapy regimens should compared in the future.

Interestingly, we observed differences in eight mass spectra when we compared patients with good and poor responses. The two peptide peaks in our model, m/z 2,142.12 and m/z 3,316.19, were fragments of glutamine-fructose-6-phosphate aminotransferase and uridylate kinase. In this context, glucosamine-6-phosphate synthase is the first and rate-limiting enzyme in the hexosamine biosynthetic pathway, and uridylate kinases (also known as UMP kinases) are key enzymes in the synthesis of nucleoside triphosphates. Ours is the first study to identify these enzymes as potential tumor markers, and we speculate that higher levels of these enzymes might correlate with sensitivity to pemetrexed/platinum because all eight peaks were downregulated in patients who experienced poor treatment responses. However, further studies are needed to confirm whether these biomarkers are valid, sensitive, and specific.

In conclusion, the results of this study demonstrate that patients with advanced lung adenocarcinoma and different responses to pemetrexed plus platinum-based chemotherapy have distinct serum peptidome profiles. Specifically, we constructed a noninvasive, highly sensitive, and high-throughput predictive peptide model to predict the clinical outcomes of this patient population in response to the indicated chemotherapy regimen. The results from our validation set suggest that this predictive peptide model can accurately and reliably discriminate chemosensitive patients and thus could be useful as a tool for the clinical selection of patients who would benefit from this regimen. However, further studies are needed to confirm the clinical value of our model.

## Supporting information

S1 Dataset (XLSX)
